# *In meso* crystallogenesis. Compatibility of the lipid cubic phase with the synthetic digitonin analogue, glyco-diosgenin

**DOI:** 10.1107/S1600576720002289

**Published:** 2020-03-25

**Authors:** Leendert van Dalsen, Dietmar Weichert, Martin Caffrey

**Affiliations:** a Membrane Structural and Functional Biology Group, School of Medicine and School of Biochemistry and Immunology, Trinity College Dublin, Dublin D02 R590, Ireland

**Keywords:** *in meso* method, membrane protein, mild non-ionic detergent, small-angle X-ray scattering, X-ray crystal structure

## Abstract

Digitonin has long been used as a mild detergent for extracting proteins from membranes for structure and function studies. As supplied commercially, digitonin is inhomogeneous and requires lengthy pre-treatment for reliable downstream use. Glyco-diosgenin (GDN) is a recently introduced synthetic surfactant with features that mimic digitonin. It is available in homogeneously pure form. GDN is proving to be a useful detergent, particularly in the area of single-particle cryo-electron microscopic studies of membrane integral proteins. With a view to using it as a detergent for crystallization trials by the *in meso* or lipid cubic phase method, it was important to establish the carrying capacity of the cubic mesophase for GDN. This was quantified in the current study using small-angle X-ray scattering for mesophase identification and phase microstructure characterization as a function of temperature and GDN concentration. The data show that the lipid cubic phase formed by hydrated monoolein tolerates GDN to concentrations orders of magnitude in excess of those used for membrane protein studies. Thus, having GDN in a typical membrane protein preparation should not deter use of the *in meso* method for crystallogenesis.

## Abstract 2

The lipid cubic phase is compatible with the synthetic digitonin analogue, GDN, over a considerable range of concentrations, indeed well beyond those likely to be encountered in typical membrane crystallization studies. Thus, based on its compatibility with the cubic phase, there is every reason to use GDN as a detergent for preparing membrane proteins that are subsequently subjected to *in meso* crystallogenesis.

## 1. Introduction   

**Figure 1:**
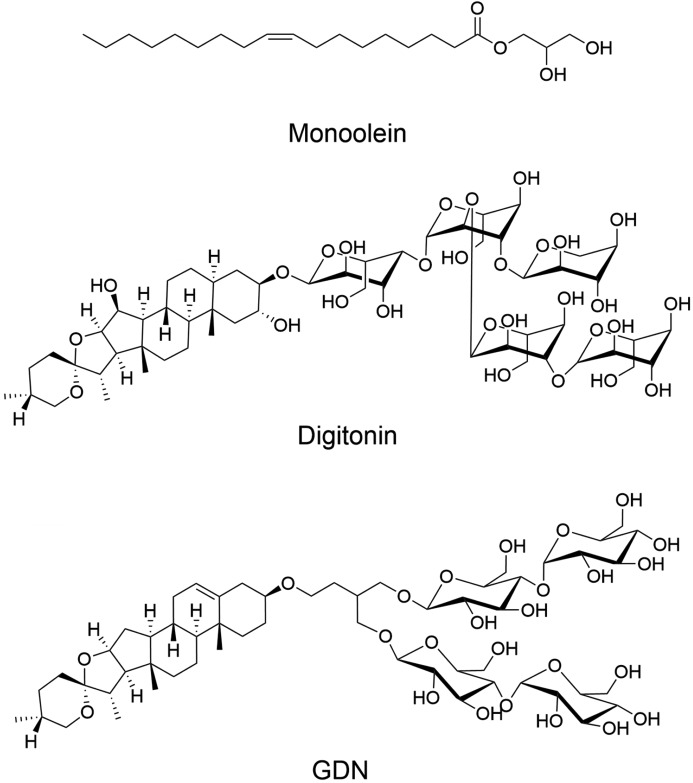
Chemical structures of monoolein, digitonin and GDN.

The foxglove plant, *Digitalis purpurea*, is the source of useful natural products that include digitalin and digitonin. Digitalin is a mixture of cardiac glycosides used to treat heart conditions (Whayne, 2018 ▸). Digitonin is a steroidal saponin used extensively as a mild non-ionic detergent (Fig. 1 ▸) (Bridges, 1977 ▸). Digitonin also finds application as a cholesterol precipitating agent (Haust *et al.*, 1966 ▸). It has long been used in membrane protein structure and function studies, where it serves to provide a hospitable micellar environment for fragile membrane integral proteins and complexes (Weigel *et al.*, 1983 ▸). However, as supplied commercially, digitonin is expensive, of variable quality, and requires lengthy and often repeated pre-treatments that include boiling and a week-long incubation followed by filtration and lyophilization (Bridges, 1977 ▸). Delivering membrane proteins of consistently high quality and stability using digitonin has generally been considered a challenge.

A synthetic alternative to digitonin, glyco-diosgenin (GDN), was introduced in 2012 (Fig. 1 ▸) (Chae *et al.*, 2012 ▸). Chemically, this amphiphile consists of a rigid steroid-type diosgenin hydro­phobic segment and a hydro­philic di-maltose head group. GDN has grown in popularity and is now used as a digitonin analogue in membrane protein structure and function studies. Increasingly, the detergent is being employed for single-particle cryo-electron microscopy (cryoEM) studies of membrane proteins. Several high-profile structures were published recently where GDN was included in the cocktail used to deliver the proteins for cryoEM characterization (Zhang *et al.*, 2017 ▸; Krishna Kumar *et al.*, 2019 ▸; Walter *et al.*, 2019 ▸; Xu *et al.*, 2019 ▸).

For reasons of cost and quality variability, digitonin is not commonly used as a detergent with which to perform membrane protein crystallization trials. However, with the success GDN has had as an analogue of digitonin, particularly for cryoEM work, consideration is being given to using it in membrane protein crystallogenesis.

**Figure 2:**
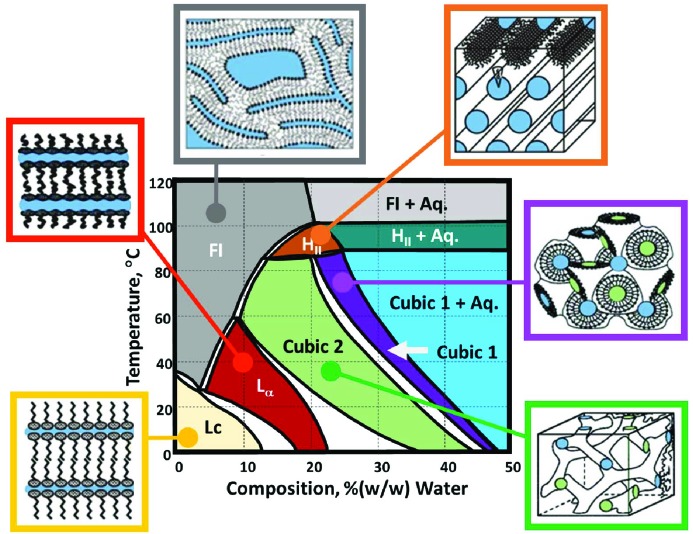
Phases formed by monoolein as a function of temperature and hydration. At the centre of the figure is the corresponding temperature–composition phase diagram. Arrayed around it are cartoon representations of the solid (Lc), liquid (FI) and liquid crystalline [cubic 1 (*Pn*3*m*), cubic 2 (*Ia*3*d*), H_II_, L_α_) phases, where coloured zones correspond to water layers or channels. The phase diagram was generated in the heating and cooling directions from 20°C. Liquid crystalline phases below ∼17°C are in an undercooled thermodynamically unstable state. Adapted from Caffrey & Cherezov (2009 ▸).

The *in meso* crystallization method employs a bicontinuous lipid cubic phase as a medium in which to reconstitute membrane proteins as a prelude to crystal growth (Fig. 2 ▸) (Landau & Rosenbusch, 1996 ▸; Caffrey, 2015 ▸). It has been used with a range of membrane proteins from transporters and channels to enzymes and receptors. To date, the *in meso* method is responsible for over 740 records in the Protein Data Bank (Burley *et al.*, 2018 ▸; https://www.rcsb.org). Crystallization by the *in meso* method takes place in a liquid crystal, also known as a mesophase. While the cubic mesophase is compatible with a host of adventitious materials that accompany the protein into the crystallization mix, it has its limits. Thus, for example, detergents and lipids, at too high a concentration, can cause the cubic phase to transition entirely to the lamellar liquid crystalline (L_α_) phase (Ai & Caffrey, 2000 ▸; Cherezov *et al.*, 2002 ▸; Misquitta & Caffrey, 2003 ▸; Ma *et al.*, 2017 ▸). Unfortunately, the L_α_ phase, in isolation, does not support the growth of macroscopic 3D crystals of the type needed for structure determination by means of macromolecular X-ray crystallography (Caffrey, 2008 ▸).

The possibility of using membrane proteins prepared with GDN for *in meso* crystallization has been discussed within the community. The goal of the current study is to answer the question, How compatible is the lipid cubic phase with GDN? This was approached by monitoring the phase behaviour of hydrated monoolein (Fig. 1 ▸), the mono­acyl­glycerol lipid most commonly used for *in meso* crystallogenesis, as a function of GDN concentration. Small-angle X-ray scattering (SAXS) was used for phase identification and for phase microstructure characterization.

The results show that the cubic phase of monoolein is compatible with GDN over a considerable range of concentrations, indeed well beyond those likely to be encountered in typical membrane crystallization studies. Thus, on the basis of its compatibility with the cubic phase formed by hydrated monoolein, there is every reason to use GDN as a detergent for preparing membrane proteins that are subsequently subjected to *in meso* crystallogenesis.

## 2. Experiments   

### 2.1. Materials   

Monoolein (356.54 g mol^−1^) was purchased at >99% purity (Nu Chek Prep Inc., Elysian, MN, USA, catalogue No. M-239; Lot M239-O25-C) and was used without further purification. Purity was confirmed by thin-layer chromatography as described by Misquitta & Caffrey (2001 ▸). Glyco-diosgenin (GDN101, 1165.31 g mol^−1^) was obtained from Anatrace (Maumee, OH, USA) at a reported purity of ≥98% and was used without purification.

### 2.2. Sample preparation   

Samples for phase diagram construction were prepared at 60%(*w*/*w*) monoolein and 40%(*w*/*w*) aqueous GDN solutions using a coupled-syringe mixing device as described elsewhere (Cheng *et al.*, 1998 ▸; Ai & Caffrey, 2000 ▸). The homogeneous samples were transferred to 1 mm-diameter glass X-ray capillaries (HR6-104, Hampton Research Corporation, Aliso Viejo, CA, USA) using a 100 mm-long, 22 gauge, end point style 3 needle (Hamilton Company, Reno, NV, USA, part No. 7804-01) and centrifuged (Eppendorf centrifuge 5810 R, Eppendorf, Hamburg, Germany) at 40°C and 4000 r min^−1^ to the bottom of the capillary. Loaded capillaries were flame sealed, glued with 5 min ep­oxy (Araldite Rapid Ep­oxy Adhesive, Pearse Street Hardware Ltd, Dublin, Ireland) and stored at 20°C for up to four days prior to data collection.

### 2.3. X-ray diffraction   

Diffraction measurements were made at the Swiss Light Source (SLS) MS-Powder beamline using a 12.6 keV (0.984 Å) beam measuring 0.1 mm wide and 0.4 mm high at the sample. The incident flux was ∼4 × 10^10^ photons s^−1^ and the exposure time was 10 s. Diffraction data were recorded using a 1D Mythen detector. The sample was rotated along its long axis continuously at one revolution per second during data collection. A fresh unexposed part of the sample was used for each measurement by translating the capillary 0.2 mm along the capillary axis between measurements. Temperature was controlled using an Oxford Cryosystem at a dry nitro­gen gas flow rate of 5 l min^−1^ in the heating range from 23°C (ambient temperature at the SLS) to 40°C and in the cooling direction from 23 to 0°C, covering the range that might be used for crystallization trials (Caffrey & Cherezov, 2009 ▸). The average temperature change rate was 1°C min^−1^. Intensity versus scattering angle (*I*–2θ) plots of the diffraction data were analysed for phase identification and lattice parameter determination as described elsewhere (Caffrey, 1987 ▸; Ai & Caffrey, 2000 ▸).

Of note is the fact that the phase behaviour of the current hydrated monoolein system is complex, and slight changes in temperature and/or composition induce changes in phase state and lattice parameter (Qiu & Caffrey, 2000 ▸). As reported previously, lattice parameters are sensitive to the lot number of commercial monoolein. Thus, we found that the microstructure of a reference hydrated lipid sample varied from 101 to 106 Å at 20°C (Misquitta & Caffrey, 2003 ▸). The error associated with the cubic *Pn*3*m* phase lattice parameter value is ±2.5 Å. The origin of this variation is not known. As noted above, we have tested the monoolein as supplied by the manufacturer and it is >99% pure, as judged by thin-layer chromatography.

## 3. Results   

The goal of this study was to determine the carrying capacity of the cubic mesophase formed by hydrated monoolein for the detergent GDN. By ‘carrying capacity’ is meant the extent to which GDN can be included in the mesophase before it transitions completely to the lamellar phase where crystallization by the *in meso* method is not possible (Caffrey, 2008 ▸). Samples were prepared in the way they would be for membrane protein crystallization trials by mixing molten monoolein with aqueous solution in a weight ratio of 3/2. The aqueous solution contained GDN over a range of concentrations up to 100 m*M* [11.65%(*w*/*v*)]. This is orders of magnitude above (i) the critical micelle concentration (CMC) of GDN at 18 µ*M* [0.0021%(*w*/*v*); Chae *et al.*, 2012 ▸] and (ii) the concentration that is likely to be used with membrane proteins for cryoEM or crystallization studies (Zhang *et al.*, 2017 ▸; Krishna Kumar *et al.*, 2019 ▸; Walter *et al.*, 2019 ▸; Xu *et al.*, 2019 ▸). SAXS was used for mesophase identification and for microstructure characterization. Measurements were made in the heating direction from room temperature (23°C; see Section 2.3) to 40°C and in the cooling direction from 23 to 0°C to mimic conditions likely to prevail during crystallization trials.

The thermotropic and lyotropic phase properties of monoolein in combination with water have been studied extensively (Fig. 2 ▸) (Briggs *et al.*, 1996 ▸; Qiu & Caffrey, 2000 ▸). Temperature–composition phase diagrams have been mapped out from 0 to 110°C and from 0 to 60%(*w*/*w*) water. From these investigations, it is known that, at room temperature (∼20°C) and at or close to full hydration, the cubic* Pn*3*m* and cubic *Ia*3*d* phases are stable. The cubic *Pn*3*m* phase persists to ∼90°C, at which point it transitions to the inverted hexagonal, H_II_, phase. Under equilibrium conditions, the solid lamellar crystal (Lc) phase is stable below ∼17°C. However, the cubic phase formed at room temperature is prone to undercooling. Thus, upon lowering the temperature from ambient to 0°C, the cubic phase persists in a long-lived metastable state, as illustrated in Fig. 2 ▸. This undercooling feature is used to advantage for performing crystallization trials with thermally labile proteins (Misquitta *et al.*, 2004 ▸; Caffrey, 2015 ▸).

**Figure 3:**
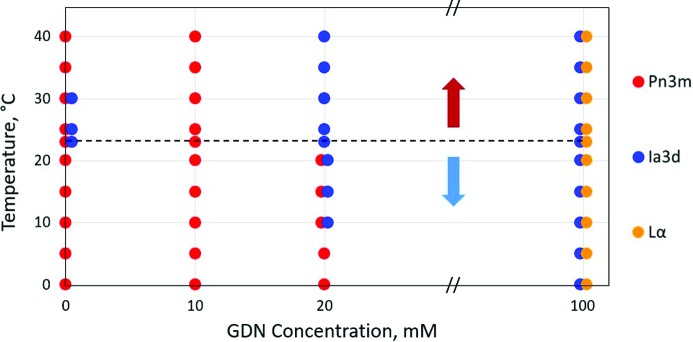
Phase behaviour of hydrated monoolein as influenced by GDN concentration and by temperature. Phase identification was based on SAXS measurements recorded in the heating direction from 23 to 40°C (red arrow) and in the cooling direction from 23 to 0°C (blue arrow). Samples were prepared with monoolein and aqueous GDN solutions in a 3/2 weight ratio. The dashed horizontal line identifies ambient temperature, 23°C.

These well established characteristics of hydrated monoolein were reproduced in the current study. Thus, along the 0 m*M* GDN isopleth (line of constant composition) in the phase diagram shown in Fig. 3 ▸, cubic phases were observed in the heating direction from 23 to 40°C and in the cooling direction from 23 to 0°C. Adding GDN to the system at a concentration of 10 m*M* had minimal effect on phase behaviour. At 20 m*M* GDN, the pure cubic *Pn*3*m* phase was stable below 10°C. It coexisted with the cubic *Ia*3*d* phase in the 10 to 23°C range. At and above 23°C, the cubic *Ia*3*d* phase alone was stabilized. Increasing GDN concentration to its reported solubility limit of 100 m*M* GDN gave expression to the L_α_ phase, which coexisted with the cubic *Ia*3*d* phase over the entire temperature range from 0 to 40°C. Along this isopleth, the L_α_ phase dominated at low temperatures. At high temperatures, the cubic *Ia*3*d* phase was the major phase (Figs. 3 ▸ and 4 ▸).

**Figure 4:**
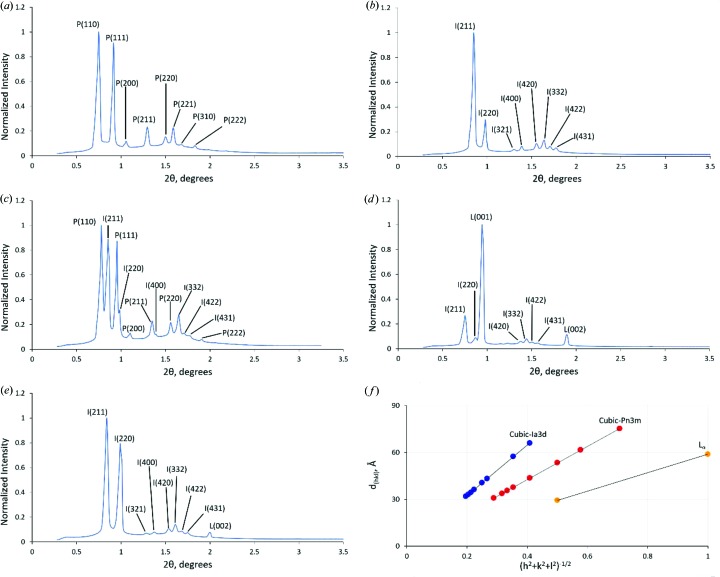
Small-angle X-ray scattering from mesophases formed by hydrated monoolein at different temperatures and GDN concentrations. Data are shown as scattered X-ray intensity (normalized to the highest value in the plot) versus scattering angle (2θ) in degrees. (*a*) Cubic *Pn*3*m* phase (P) at 20°C and 10 m*M* GDN. (*b*) Cubic* Ia*3*d* (I) phase at 30°C and 20 m*M* GDN. (*c*) Cubic *Pn*3*m*/cubic *Ia*3*d* phase coexistence at 15°C and 20 m*M* GDN. (*d*) Cubic *Ia*3*d*/L_α_ phase coexistence at 0°C and 100 m*M* GDN. L_α_ (L) is the dominant phase. (*e*) Cubic *Ia*3*d*/L_α_ phase coexistence at 35°C and 100 m*M* GDN. The cubic *Ia*3*d* phase dominates. (*f*) Phase identification, indexing and lattice parameter determination is illustrated for data in panels (*a*), (*b*) and (*d*). Key: *d*_(*hkl*)_, experimentally determined *d*-spacing value; *h*, *k*, *l*, Miller indices of Bragg reflections. The slope of the line of best fit is the lattice parameter of the phase with corresponding values of 162.1, 106.7 and 59.6 Å for the cubic *Ia*3*d*, cubic *Pn*3*m* and L_α_ phases, respectively.

Exemplar SAXS *I*/2θ data, recorded as part of this study and used for phase identification and phase microstructure characterization, are included in Fig. 4 ▸. The lattice parameters observed in this study are similar to those reported previously (Briggs & Caffrey, 1994 ▸; Qiu & Caffrey, 2000 ▸) (Table 1 ▸).

As with most detergents, GDN is typically used in work on membrane proteins at concentrations that are a few multiples of its CMC value. Accordingly, these are the concentrations likely to be used in crystallization trials when the solubilized protein solution is combined with monoolein for cubic phase formation. If the protein is concentrated with spin-concentrators, for example, or by the cubicon method (Ma *et al.*, 2017 ▸), the concentration of detergent can rise in parallel with protein concentration. However, in such circumstances, it is unlikely that GDN concentration will rise to more than ten times the concentration used for solubilization. A rough estimate therefore puts an upper limit on the GDN concentration likely to be encountered in crystallization trials at 0.5 m*M*. The data in Fig. 3 ▸ show that it is not until GDN levels rise above 20 m*M* that the cubic phases are destabilized. This result should provide comfort to those using GDN as a detergent with membrane proteins. Thus, under normal conditions, such solutions are compatible with *in meso* crystallogenesis. Even at a GDN concentration of 100 m*M* the cubic *Ia*3*d* phase persists and remains in coexistence with the L_α_ phase. Indeed, under these conditions, the *Ia*3*d* phase dominates over the L_α_ phase at higher temperatures and may well prove compatible with *in meso* crystallogenesis (Caffrey, 2008 ▸).

## 4. Discussion   

The concentration of monoolein in the cubic phase at full hydration and ambient temperature is extremely high at approximately 2 molar. From the data in Fig. 3 ▸, we see that GDN does not destabilize the cubic phase over the full temperature range examined until its concentration goes above 20 m*M*. This corresponds to the presence in the mesophase of one GDN molecule for every 200 molecules of lipid. The lamellar phase coexists with the cubic *Ia*3*d* phase at 100 m*M* GDN, corresponding to one detergent molecule for every 40 molecules of lipid. These are useful figures to bear in mind when working with the lipid cubic phase either as a medium in which to perform crystallogenesis or as a membrane mimetic for structure–function work (Li & Caffrey, 2020 ▸). They attest to the ability of this remarkable nanoporous biomaterial to accommodate considerable quantities of amphiphilic substances which, at high enough concentrations, will completely solubilize it.

It is worth reflecting on the mechanism by which GDN, at higher concentrations, might destabilize the cubic phase. Monoolein has a small polar head group and a relatively large apolar segment. In the cubic mesophase, it adopts what has been referred to as a dynamically averaged wedge shape (Cherezov *et al.*, 2002 ▸; Caffrey, 2008 ▸). Close packing of monoolein molecules of this form gives rise to a mesophase that includes a pair of highly curved polar/apolar membrane interfaces. In distinct contrast, the GDN molecule has a relatively small and rigid steroid-like apolar nucleus and a bulky hydrated polar head group (Fig. 1 ▸). Roughly speaking, GDN can be described as having a dynamically averaged conical shape that is opposite or possibly complementary in a geometric sense to that of monoolein. Specifically, the polar head group of GDN is large and bulky; in monoolein, it is small. Thus, titrating GDN into a cubic mesophase made of monoolein is expected to lower curvature at the polar/apolar interface, thereby making the bilayered membrane more planar. At high enough GDN loading, the lamellar phase is expected to stabilize and to become the dominant phase state, as was observed (Fig. 3 ▸). This interface flattening effect is enhanced at lower temperatures where the acyl chain of monoolein has a reduced number of *trans*/*gauche* isomerizations along its length and the molecule is naturally less wedge and more cylindrically shaped, which favours lamellar phase formation (Qiu & Caffrey, 2000 ▸). The rigid steroid-like nucleus of GDN is likely to act on bilayer fluidity in the same way that cholesterol does, by imposing order on adjacent lipid acyl chains in the membrane. This too will contribute to stabilizing the lamellar phase (Cherezov *et al.*, 2002 ▸).

GDN is a synthetic detergent that has only recently become available. Alongside it, there are dozens of other detergents that are used on a regular basis by the membrane protein structural and functional biology community. The compatibility of several of these detergents with the lipid cubic phase has been assessed, as described here for GDN. For the most part, the detergents investigated, which include alkyl glycosides, alkyl neo­pentyl-glycols, lauryl di­methyl­amine-*N*-oxide, sodium do­decyl sulfate and octa­ethyl­ene glycol monodo­decyl ether, are tolerated by the cubic phase at low concentrations (Ai & Caffrey, 2000 ▸; Misquitta & Caffrey, 2003 ▸; Ma *et al.*, 2017 ▸; Zatloukalová *et al.*, 2018 ▸). However, given the general dynamically averaged conical shape of these surfactants, at high concentrations unsurprisingly they can destabilize the cubic phase in favour of the lamellar phase. Fortunately, the concentrations at which these are used in structure determination work are such that they are compatible with the *in meso* crystallogenesis method.

## 5. Conclusion   

GDN is an attractive mild non-ionic detergent for membrane protein structure and function work. It is proving itself in the cryoEM field, where it has been associated with the structure determination of a number of high-visibility membrane proteins and complexes. The results of the current study show that GDN, at concentrations typically used in membrane protein studies, is entirely compatible with the lipid cubic phase. Accordingly, if a membrane protein proves stable and functional in GDN, it can proceed into crystallization trials by the *in meso* method in the knowledge that adventitious detergent should not destabilize the mesophase in which crystals of high-resolution structure quality will be encouraged to grow.

## Figures and Tables

**Table 1:** Microstructure of the phases formed by hydrated monoolein as influenced by GDN concentration and temperature Tables on the left and right refer to data collected from 23°C in the cooling and heating directions, respectively. Lattice parameter calculation was based on analysis of the SAXS patterns recorded in the cooling direction from 23 to 0°C and in the heating direction from 23 to 40°C (see Fig. 4 ▸). Samples were prepared with three parts monoolein and two parts aqueous GDN solution.

		Lattice parameters (Å)						Lattice parameters (Å)		
Temperature (°C)	GDN concentration (m*M*)	Cubic *Pn*3*m*	Cubic *la*3*d*	L_α_		Temperature (°C)	GDN concentration (m*M*)	Cubic *Pn*3*m*	Cubic *la*3*d*	L_α_
23	0	102.3	162.2			23	0	102.1		
23	10	106.8				23	10	106.1		
23	20	102.1	162.3			23	20		163.4	
						23	100		173.9	57.7
20	0	102.3				25	0	102.2	163.3	
20	10	106.7				25	10	106.1		
20	20	102.6	162.1			25	20		163.3	
20	100		175.7	58.0		25	100		173.5	57.5
15	0	102.1				30	0	101.4	163.3	
15	10	106.4				30	10	105.3		
15	20	102.5	161.4			30	20		162.1	
15	100		177.5	58.3		30	100		171.0	57.1
10	0	101.6				35	0	99.8		
10	10	106.0				35	10	102.2		
10	20	102.4	160.8			35	20		160.9	
10	100		179.3	58.8		35	100		164.3	56.7
5	0	101.4				40	0	94.5		
5	10	105.7				40	10	95.0		
5	20	102.1				40	20		159.6	
5	100		181.4	59.2		40	100		160.8	56.5
0	0	101.1								
0	10	105.4								
0	20	102.0								
0	100		183.8	59.6						
